# Gastric-type Mucinous Carcinoma with an Abnormal Increase of CA199: A Case Report and Literature Review

**DOI:** 10.3389/fsurg.2022.945984

**Published:** 2022-07-04

**Authors:** Xue-qian Qian, Fen-fen Wang, Yun Liang, Li-Li Chen, Xiao-yun Wan

**Affiliations:** Department of Gynecologic Oncology, Women's Hospital, School of Medicine, Zhejiang University, Hangzhou, China

**Keywords:** cervical adenocarcinoma ***in situ***, cervical adenocarcinoma, gastric-type mucinous carcinoma (GAS), CA199, cervical cancer

## Abstract

**Objective:**

Gastric-type mucinous carcinoma (GAS), as a rare subtype of mucinous adenocarcinoma, accounts for approximately 1%–3% of cervical adenocarcinoma. It was considered as a new type of cervical mucinous adenocarcinoma by the World Health Organization (WHO) in 2014. GAS represents more aggressive disease than does usual type endocervical adenocarcinoma (UEA).

**Case report:**

A case of cervical adenocarcinoma with an abnormal increase of CA199 in a 50-year-old Chinese woman was reported. Our patient presented with abnormal vaginal discharge and combined with elevated Ca199 at the value of 2,729 U/mL. Imaging examinations showed no abnormalities. Diagnostic conical resection suggested cervical adenocarcinoma *in situ*. Post-operative pathology confirmed mucinous cervical adenocarcinoma (considering gastric type), infiltrating cervical interstitial >2/3, involving the deep myometrium, accompanied by vascular carcinoma infiltration and lymph node metastasis.

The patients received an extensive hysterectomy and post-operative adjuvant chemoradiotherapy. The chemotherapy regimen was paclitaxel, combined with platinum. After 20 months of follow-up, the patient showed no signs of recurrence.

**Conclusion:**

Preoperative diagnosis of cervical adenocarcinoma is insidious and can be easily misdiagnosed. For patients with high preoperative Ca199, the possibility of GAS should be kept open.

## Introduction

Cervical cancer is the fourth most common cancer among women, with an estimated 570,000 new cases in 2018, accounting for 6.6% of all female cancer cases worldwide (1). Cervical adenocarcinoma (AC) accounts for about 3%–6% of cervical malignant tumors, and its incidence has been increasing year by year in recent years (2–5). Most studies suggest that the overall survival rate of patients with AC after surgical treatment is lower than that of patients with squamous cell carcinoma (SCC). The 5-year overall survival (OS) of SCC is 58.6%–85.2%, and the 5-year OS of AC is 26.7%–75.4% (6, 7). The treatment of choice for most women with early-stage AC is radical hysterectomy. However, patients with late stage should also receive primary radiation with chemotherapy (8).

At present, preoperative diagnosis of cervical adenocarcinoma is still difficult, lack of specific tumor markers, and lesions tend to grow toward the cervical canal, so it is easy to be ignored. Gastric-type mucinous carcinoma (GAS) is a special type of cervical mucinous adenocarcinoma, which often indicates poor prognosis and rapid progression (9–12). It is an HPV-unrelated form of cervical cancer. Clinically, it has a poorer prognosis than usual-type AC, with an unusual pattern of spread, including lymphatic spread and invasion of the parametrial space, resulting in poorer progression-free survival (PFS) and OS (13). Most studies have shown that serum elevation of CA199 is useful in the diagnosis of upper gastrointestinal adenocarcinoma, and its greatest sensitivity is in the detection of pancreatic adenocarcinoma (14, 15). To improve clinicians’ awareness of the disease, here, we report an abnormally elevated preoperative CA199 in a patient who was not correctly diagnosed preoperatively. This case report was approved by the Ethics Committee of Women's Hospital, School of Medicine, Zhejiang University. The informed consent form was signed by the patient. The pathology was confirmed by the Department of Pathology, Women's Hospital, School of Medicine, Zhejiang University.

## Case Presentation

A case of cervical adenocarcinoma with an abnormal increase of CA199 in a 50-year-old Chinese woman is reported. There is no family history of the disease. The clinical features of the patient at baseline are summarized in Table 1. The follow-up time is 20 months.

**Table 1:** The clinical features of the patient.

Clinical features of the patient	
Age (years)	48(y)
Gravida(times)	4
Para(times)	2
Family history	No
Vaginal drainage	Present
Postcoital bleeding	Present
Tumor marker	
CA125	Normal
CA199	2729 U/mL
CEA	Normal
Lymph node metastasis	Present
Ovary metastasis	Present
FIGO Stage	IIIC1
Cytology	Negative
HPV infection	Negative
Deep stromal invasion	Present
Lymph vascular space invasion	Present
Surgical approach	Abdominal surgery
Adjuvant therapy	Chemotherapy Radiotherapy

**Figure 1:**
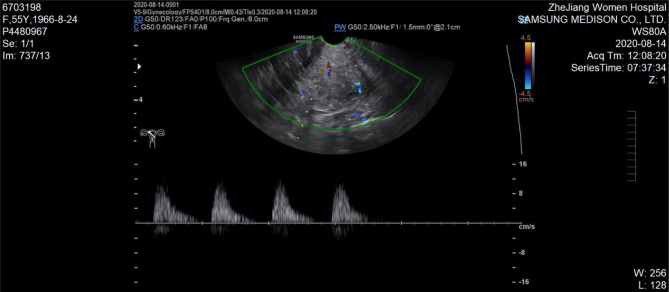
Ultrasonography showed echogenicity of thickened myometrium, suggesting adenomyosis.

Our patient presented with abnormal vaginal discharge. Gynecological examination showed no obvious abnormal lesion in the cervix. Cervical cytology revealed atypical adenosine cells and the HPV test was negative. Laboratory examination found an abnormal elevation of Ca199 at a high value of 2,729 IU/ mL. Ultrasonography showed an echogenicity of thickened myometrium, suggesting adenomyosis (see Figure 1). Imaging examinations including MRI and abdominal CT showed no obvious cervical lesions. Considering that CA199 was unusually high, PET-CT was done before surgery, which could rule out distant metastasis or other diseases. However, the results only suggested adenomyosis. Lastly, cervical biopsy revealed adenocarcinoma *in situ*.

**Figure 2:**
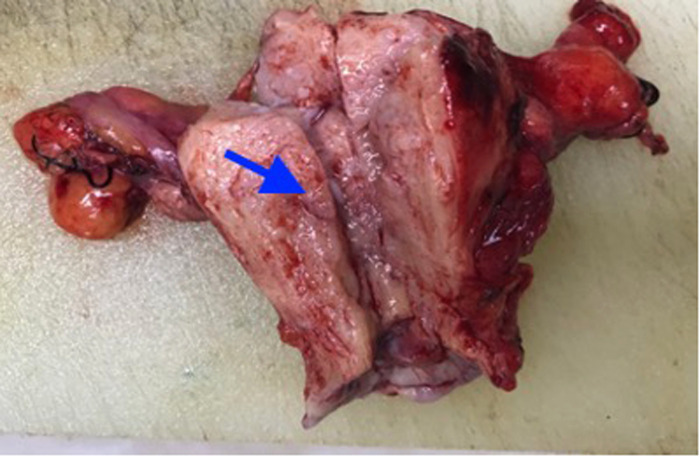
The anterior and posterior walls of the uterus are diffusely thickened, with localized soft, fish-like tissue.

**Figure 3:**
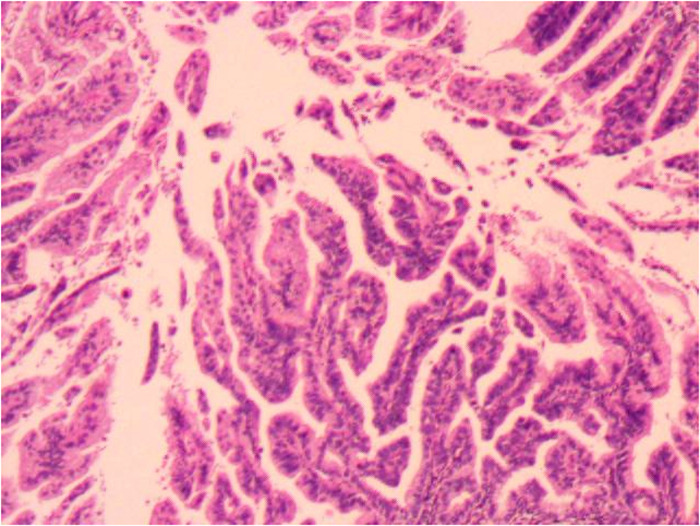
Cervical gastric adenocarcinoma at high magnification. This pathological image shows three characteristics: (1) abundant cytoplasm; (2) Clear boundaries between cells; (3) Typical cystic and tubular glands are presented.

**Figure 4:**
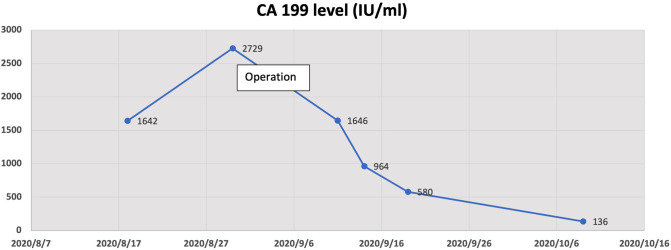
The change graph of the Ca199 value showed that it gradually decreased after operation, suggesting a correlation between elevated CA199 and cervical adenocarcinoma.

Considering the unsatisfactory visualization at the time of colposcopy, the patient underwent a diagnostic conical resection, which was also suggestive of cervical adenocarcinoma *in situ*. In addition, gastroenterostomy indicated normal results. Hysterectomy was not the only treatment option because local resection also offered a possible option. We talked to the patient about the pathologic findings and an unexplained high elevated ca199 value, and the patient chose hysterectomy. Intra-operative rapid freezing suggested uterine adenocarcinoma and cervical focal adenocarcinoma. An extensive hysterectomy and pelvic lymph node resection were eventually performed. Post-operative pathology confirmed mucinous cervical adenocarcinoma (considering gastric type, Figures 2, 3), infiltrating cervical interstitial >2/3, involving the deep myometrium and accompanied by vascular carcinoma infiltration and lymph node metastasis. Ascites cytology was negative. The patient’s final stage was stage IIIC1. In addition, patients received post-operative adjuvant chemoradiotherapy. The chemotherapy regimen was paclitaxel, combined with platinum. Immunohistochemical results showed positive protein molecules such as Ki-67, CK7, CEA, and CA199, suggesting the diagnosis of GAS. The Ca199 value showed that it gradually decreased to normal after operation, suggesting a correlation between elevated CA199 and cervical adenocarcinoma (Figure 4).

## Disscussion

GAS was included in the WHO classification in 2014 (16). The first report in English was published by McKelvey and Goodling in 1963. These tumors were almost always fatal and resistant to common treatments (17). Shin Nishio et al. reported that among the 328 enrolled patients with endocervical adenocarcinoma, a total of 95 of the 328 tumors were classified as GAS. Compared with UEA, GAS was more significantly associated with bulky mass, deep stromal invasion, lymph vascular space invasion, parametrial invasion, ovarian metastasis, and positive ascitic fluid cytology (18). Our patient also displayed a great deal of aggressiveness and was pathologically confirmed vascular- and lymphatic-positive.

The main clinical manifestations are vaginal mucous or watery secretions, but also irregular vaginal bleeding. Cytological screening and HPV detection are of little value in the diagnosis of cervical adenocarcinoma (19). Therefore, for patients with multiple vaginal discharge but no positive findings of HPV and cytology, they should be alert to the possibility of GAS. MRI, deep biopsy, or conical incision is necessary. In our case, vaginal fluid was the main manifestation. Preoperative HPV examination showed negative results. Although cervical biopsy and diagnostic coning were performed, neither was confirmed. This further confirms the difficulty of early diagnosis of the disease.

The serum level of squamous cell carcinoma antigen (SCC) is considered to be a representative marker of cervical cancer. Tumor markers such as CA199 and CA 125 are closely correlated with gynecological malignant tumors, but they are mainly increased in ovarian malignant tumors and have poor correlation with common type cervical cancer (20). Through our case, we hope that clinicians become aware of those patients with a high preoperative CA199 and keep the possibility of GAS open. Koprowski and colleagues first identified the CA 199 antigen, which has since been widely used to investigate and treat patients with pancreatic cancer (21). CA199 antigen mainly exists as epitopes on glycolipids and salivary lactate -N fucopeptide II ganglioside in tissues. It is synthesized from normal human pancreas and bile duct cells, as well as the epithelium of the stomach, colon, and endometrium. Therefore, CA199 can be elevated in many types of gastrointestinal cancer such as colorectal cancer, esophageal cancer, and hepatocellular cancer. Moreover, CA199 antigen can also be elevated in ovarian mucinous neoplasms (22). For perimenopausal women patients, it is also necessary to make a differentiation with regard to endometrial cancer, uterine fibroids, inflammatory conditions, or diseases other than cancers (23, 24). Therefore, the medical condition must be comprehensively analyzed in combination with other serological and imaging indexes. Moreover, Abe et al. reported that the 5-year-survival rates of sr-CA199-positive patients were poorer than those of the negative ones (*P* < 0.1) (25). Our patient has been followed up only for 20 months, and so far, there has been no sign of recurrence, but a further extension of follow-up time is needed to determine the correlation between the CA199 level and the survival rate.

For the treatment of GAS, due to the small number of cases, treatment experience still needs to be accumulated. In this case, the pathology of rapid intraoperative freezing indicated uterine and cervical adenocarcinoma, so the scope of surgery was expanded, and extensive hysterectomy and post-operative adjuvant chemoradiotherapy were performed. It is worth mentioning that in our case, adequate preoperative communication and the patient’s perspective choice are very important, when preoperative pathology only indicates cervical adenocarcinoma *in situ*. Although hysterectomy is recommended first for patients without any fertility requirements, a sufficiently extensive local resection of the lesion is also feasible.

GAS has a low incidence and poor overall prognosis. How to find effective methods for early diagnosis and treatment is particularly important to reduce recurrence and the mortality rate of patients. Therefore, it is of great significance to find some key biological targets. Recently, a large comprehensive genomic study of 228 patients with cervical cancer reported that patients with endometrial-like cervical cancers, which mainly comprised HPV-negative tumors, had relatively high frequencies of KRAS, ARID1A, and PTEN mutations. Such molecular analyses may lead to a breakthrough, revealing new potential therapeutic targets for lethal cervical cancer, such as GAS (26).

## Conclusion

In conclusion, GAS is significantly associated with poor outcomes as well as with poorer survival outcomes, and, therefore, for patients with a high preoperative Ca199, the possibility of GAS should be kept open.

## Data Availability

The original contributions presented in the study are included in the article/Supplementary Material; further inquiries can be directed to the corresponding author/s.
